# Establishing the Embryonic Axes: Prime Time for Teratogenic Insults

**DOI:** 10.3390/jcdd4030015

**Published:** 2017-09-11

**Authors:** Thomas W. Sadler

**Affiliations:** 1 Senior Fellow, Greenwood Genetics Center, Greenwood, SC 29646, USA; tsadler@hermit-house.com; Tel.: +1-406-596-0198; 2 Department of Pediatrics, University of Utah, Salt Lake City, UT 84108, USA; 3 Department of Anatomy, Quillen College of Medicine, East Tennessee State University, Johnson, TN 37614, USA; 4 78 Lemon Gulch Lane, Sheridan, MT 59749, USA

**Keywords:** laterality, birth defects, heterotaxy, situs inversus, embryonic axes, heart defects

## Abstract

A long standing axiom in the field of teratology states that the teratogenic period, when most birth defects are produced, occurs during the third to eighth weeks of development post-fertilization. Any insults prior to this time are thought to result in a slowing of embryonic growth from which the conceptus recovers or death of the embryo followed by spontaneous abortion. However, new insights into embryonic development during the first two weeks, including formation of the anterior-posterior, dorsal-ventral, and left-right axes, suggests that signaling pathways regulating these processes are prime targets for genetic and toxic insults. Establishment of the left-right (laterality) axis is particularly sensitive to disruption at very early stages of development and these perturbations result in a wide variety of congenital malformations, especially heart defects. Thus, the time for teratogenic insults resulting in birth defects should be reset to include the first two weeks of development.

According to the principles of teratology, as defined by a founding father of that science, James Wilson, embryos begin to be susceptible to a teratogenic insult, “toward the end of the germ layer stage [when] specific organ differentiation begins” [1]. Thus, “localized defects” in particular organs, such as the heart, limbs, neural tube, etc., “cannot be predictably induced,” until the targeted structure has initiated development. Insults prior to this time have an all or none effect. That is, they result in death of the embryo or a generalized slowing of its growth from which it recovers while exhibiting no structural abnormalities [1]. For this reason graphs or charts depicting susceptible stages of embryonic development when birth defects can be induced show a peak sensitivity occurring from 3 to 8 weeks of gestation (Figure 1). Almost no defects are predicted to occur prior to the beginning of the 3rd week and a rapidly declining occurrence is observed at the conclusion of the eighth week. This 3–8 week period is the time of organogenesis when the anlage of each organ system is formed.

Recent evidence challenges this basic tenet and demonstrates that establishment of the body axes, dorso-ventral (D-V), cranio-caudal (anterior-posterior; A-P), and left-right (laterality; L-R) is essential for normal development. It is generally agreed that the first axes to form are the A-P and D-V axes. At the latest these axes are specified in the peri-implantation period soon after the blastocyst forms at approximately days 4–5 of development [2,3,4] and they may be specified even earlier [4]. By the time of implantation, the A-P axis is firmly established [4]. When establishment of the L-R axis begins also is not clear, but it is determined by positional cues provided by the A-P and D-V axes. Thus, the first step in breaking symmetry in the embryo is always related to and dependent upon orientation of the D-V and A-P axes and, therefore, signaling to establish laterality must be initiated sometime in the first week of development [5,6]. L-R differences produced when symmetry is broken are then translated into differential gene expression patterns on each side of the body. Finally, these patterns of gene expression are translated into changes in cell behavior, including proliferation rates and migration, that regulate positioning and development of organ systems [6,7].

The role of laterality signaling in normal and abnormal development has been particularly well characterized and a variety of structural birth defects involving almost every tissue and organ system has been observed when establishment of the L-R axis is disrupted (Table 1) [8,9,10,11,12,13]. The process is similar to building the framework of a house: If one side is taller than another, or too long, or off-center, then the rest of the house cannot be built without defects. The roof will not fit, or a door will not close, or the plumbing will be impossible to position properly. So it is with embryos: If the axes are not specified correctly then defects occur.

Toxic insults and/or alterations of gene expression can interfere with any of these steps from the breaking of symmetry to organ development, thereby resulting in a congenital malformation. One of the key periods in the process of establishing L-R asymmetry occurs late in the second week and early in the third week of gestation at the primitive streak stage of development when gene expression patterns important for establishing the left side are occurring [14]. At this stage no organ anlage have appeared and the embryo exists as a bilaminar disc. One of the first signaling events serving to establish the left side is the accumulation of the secreted factor nodal on the left side of the embryo. In turn nodal activates the pathway for establishing the left side that ends in expression of the transcription factor *PITX2,* a “master gene” for left sided development [15,16]. That the pathway is essential for establishing normal situs is demonstrated by experiments showing that mis-expression of *PITX2* results in laterality defects especially of the heart [17,18,19,20].

How nodal becomes restricted to the left is not clear, but evidence suggests that the neurotransmitter serotonin (5-HT) is a key signaling molecule. According to this theory, 5-HT accumulates on the right side of the embryo and represses expression of *nodal* on that side by signaling through the histone deacetylase binding partner Mad3, As a result, the concentration of nodal increases on the left side of the primitive node triggering the laterality pathway, on the left [6,21,22,23]. This hypothesis is strengthened by results showing that interference with 5-HT concentrations randomizes situs in *Xenopus* embryos [21,22]. These investigators have also demonstrated a role for the serotonin transporter SERT in laterality signaling. This finding is interesting because recent evidence suggests that antidepressants belonging to the selective serotonin reuptake inhibitor class (SSRIs) that act by inhibiting 5-HT transport via SERT may also cause congenital heart defects due to abnormal laterality signaling at these early stages of development [24].

Exposure of embryos to teratogens during this early period of development also results in laterality defects, including heart malformations. For example, exposure of rat embryos at the primitive streak stage to X-irradiation resulted in some embryos with situs inversus and others with heterotaxy, and some of these had heart malformations [25]. A similar study using trypan blue as the teratogenic agent produced almost identical results [26]. Heart defects observed in both investigations included dextrocardia, transposition of the great vessels, ventricular inversions, absent mitral and tricuspid valves, double outlet right ventricle, atrial and ventricular septal defects, tricuspid and mitral stenosis, common truncus, common atrioventricular canal, and total anomalous pulmonary venous return.

Although a wide variety of birth defects can be observed in individuals with abnormal patterns of laterality, both animal and clinical studies indicate that the most commonly affected organ is the heart. Exactly when heart development is affected is not clear, but one critical period occurs late in the second and early in the third week at primitive streak stages when heart cells are being patterned [24,27,28]. Normally, many body organs exhibit asymmetry, including the heart, blood vessels, lungs, gut tube, spleen, stomach, liver, and gall bladder. Correct positioning of thoracic and abdominal organs is called *situs solitus*. Complete reversal of all organs, where organs are reversed in a mirror image arrangement, is called *situs inversus*. Discordant organ positioning with respect to symmetry, where one or more organs are abnormally positioned (i.e., reversed in position) or if isomerisms or inversions are present, is called *situs ambiguus* or heterotaxy. Individuals with situs inversus and *situs ambiguus* are considered to have “laterality” defects [9] and these defects arise because of a failure to correctly establish left-right patterning during embryogenesis. Individuals with situs inversus do not have a high risk for having other congenital abnormalities, although they do have an increased risk for congenital heart defects [29] and their progeny are at an increased risk of having laterality disease with a greatly increased risk for complex cardiac malformations [30,31,32]. In addition, individuals with heterotaxy often have other congenital abnormalities, including a variety of midline malformations, including neural tube defects [8,9,10,11,12,31]. Furthermore, many of these individuals with midline defects will have complex congenital heart defects and many will have abnormal vascular patterns involving major vessels [13,33,34]. Interestingly, within a cohort of individuals with genetic abnormalities in their laterality signaling pathway, some will have overt heterotaxy or anomalies of organ position, while others will have isolated defects, especially heart malformations [12,13,15,33].

Thus, the most sensitive organ to be affected by abnormal L-R patterning is the heart. Virtually every type of heart defect can be observed where there is perturbation in L-R patterning signals, even in the absence of defects to other organs. Thus, l and d transpositions of the great arteries (TGAs), dextrocardia, ventricular inversions and isomerisms, atrial inversions and isomerisms, and many other congenital cardiac defects result from abnormal signaling in the laterality pathway (Table 2 [16,19,20,24,35,36]). Since most of the vasculature in an embryo begins as a bilaterally symmetrical system that then is re-patterned into the final form, vascular abnormalities, such as aortic arch defects and vena cava defects, as well as total and partial anomalous pulmonary venous return (TAPVR and PAPVR), commonly arise when abnormal laterality signaling occurs [10,12,13,37,38]. In fact abnormalities of the pulmonary vessels, like TAPVR and PAPVR, are almost pathognomonic for laterality defects and occur in over 70–80% of cases [13,33]. The types of heart and vascular defects are so varied because the heart and blood vessels are dependent upon critical L-R and A-P signaling to acquire their normal development and positioning [15,16,39,40].

An association between abnormal laterality patterning and heart defects in the absence of other congenital malformations has been observed in studies of family members with known mutations in laterality genes. In such families, a high incidence of family members exhibit only a congenital cardiac defect and no other abnormalities [12,13,15,41]. Based on these data, one of the criteria for diagnosing problems with laterality signaling and establishing the L-R axis is for a patient to have a heart malformation and have family members with heterotaxy [12,15].

As mentioned previously, it is not clear exactly when the heart becomes sensitive to insults causing abnormal laterality signaling. Certainly by the end of the second week and beginning of the third week of gestation, when the primitive streak appears and gastrulation begins, heart cells are patterned with respect to all three axes. At this time prospective heart cells migrate through the node and cranial end of the primitive streak to become arranged in a horseshoe shaped collection of cells cranial to the prospective neural plate known as the primary heart field (PHF [27,42,43]). A secondary heart field (SHF) also forms in mesoderm ventral to the pharynx. Both of these heart fields are patterned with respect to the left and right sides and craniocaudally as to their contribution to the different heart regions, including the atria, ventricles, and outflow tract [27,28,43]. This patterning is dependent upon proper signaling through the laterality pathway.

As more is learned about signaling that regulates axis patterning, it is becoming clearer that birth defects related to abnormalities in this signaling can probably be induced even earlier than the primitive streak stages described above. Evidence for this claim can be found in patients with midline abnormalities, including neural tube defects, cleft lip and palate, omphalocele, anal atresia, and stenosis, and caudal dysgenesis that occur when laterality signaling is disrupted [8,9,10,11,12,13,15,31,41]. This association between the midline and laterality is so strong that it has been estimated that if a patient has a midline malformation, such as a neural tube defect, then they are three times more likely to have a laterality issue as compared to patients without a midline defect [8,9,10,11,12]. Thus, patterning of the midline and the A-P axis is intimately linked to patterning the L-R axis [5] and it is known that patterning the A-P axis begins at least by the fourth to fifth days after fertilization [4]. In fact, some evidence suggests that both axes are beginning to be established by the two-cell stage [5]. Therefore, the graph in Figure 1 depicting the period of greatest sensitivity to teratogenic insult has been modified to indicate the sensitivity begins even earlier than the beginning of the third week after fertilization.

## Figures and Tables

**Figure 1:**
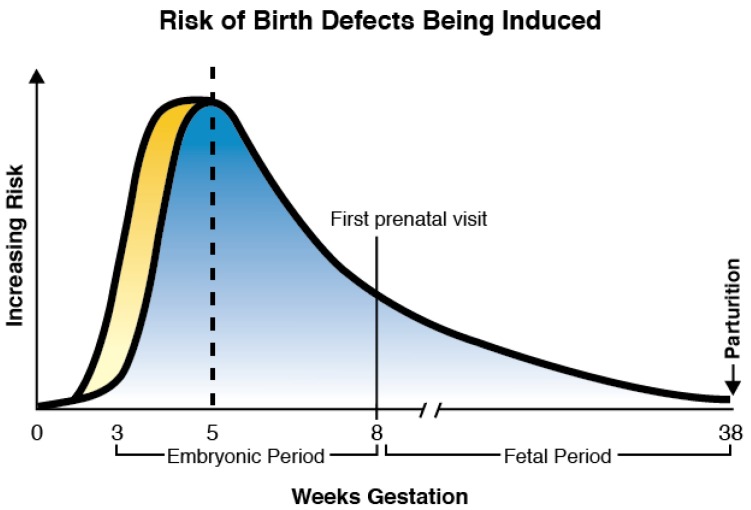
Graph showing susceptible period of teratogenesis when birth defects can be induced. Originally it was thought that the third to eighth weeks after fertilization were the most sensitive time (blue). However, recent advances in our understanding of key events regulating embryogenesis, especially those involving axes formation, has demonstrated the period of greatest susceptibility should include at least the second week and probably the first as well (yellow).

**Table 1:** Birth defects associated with abnormal laterality in humans [8,9,10,11,12,13].

Skeletal Defects	Caudal Dysgenesis
Limb defects (especially clubfoot)	Intestinal malrotation
Craniosynostosis	Kidney defects (agenesis, horseshoe)
Anal atresia	Tracheoesophageal fistula/Esophageal atresia
Imperforate anus	Genital defects
Gastroschisis	Micropthalmia
Omphalocele	Cleft lip and/or cleft palate
Neural tube defects	Vertebral anomalies

**Table 2:** Heart defects associated with abnormal laterality in humans [16,19,20,24,35,36].

Dextrocardia	Pulmonary Valve Atresia and Stenosis
Single atrium	Hypoplastic left heart syndrome
Single ventricle	Double outlet right ventricle
Transposition of great arteries	Complete atrioventricular block
Total and partial anomulous pulomary venous return	Tetralogy of Fallot
Atrioventricular canal defect	Aortic Arch defects
Endocardial cushion defects	Ventricular inversion
Atrial septal defect	Atrial inversion
Ventricular septal defect	Atrial isomerism
Coarctation of the aorta	Double inlet left ventricle
